# PROTAC-DB 3.0: an updated database of PROTACs with extended pharmacokinetic parameters

**DOI:** 10.1093/nar/gkae768

**Published:** 2024-09-03

**Authors:** Jingxuan Ge, Shimeng Li, Gaoqi Weng, Huating Wang, Meijing Fang, Huiyong Sun, Yafeng Deng, Chang- Yu Hsieh, Dan Li, Tingjun Hou

**Affiliations:** College of Pharmaceutical Sciences, Zhejiang University, Hangzhou 310058 Zhejiang, China; CarbonSilicon AI Technology Company, Ltd., Hangzhou 310018Zhejiang, China; College of Pharmaceutical Sciences, Zhejiang University, Hangzhou 310058 Zhejiang, China; College of Pharmaceutical Sciences, Zhejiang University, Hangzhou 310058 Zhejiang, China; College of Pharmaceutical Sciences, Zhejiang University, Hangzhou 310058 Zhejiang, China; Polytechnic Institute, Zhejiang University, Hangzhou 310058 Zhejiang, China; Department of Medicinal Chemistry, China Pharmaceutical University, Nanjing 210009 Jiangsu, China; CarbonSilicon AI Technology Company, Ltd., Hangzhou 310018Zhejiang, China; College of Pharmaceutical Sciences, Zhejiang University, Hangzhou 310058 Zhejiang, China; College of Pharmaceutical Sciences, Zhejiang University, Hangzhou 310058 Zhejiang, China; College of Pharmaceutical Sciences, Zhejiang University, Hangzhou 310058 Zhejiang, China; Polytechnic Institute, Zhejiang University, Hangzhou 310058 Zhejiang, China

## Abstract

Proteolysis-targeting chimera (PROTAC) is an emerging therapeutic technology that leverages the ubiquitin-proteasome system to target protein degradation. Due to its event-driven mechanistic characteristics, PROTAC has the potential to regulate traditionally non-druggable targets. Recently, AI-aided drug design has accelerated the development of PROTAC drugs. However, the rational design of PROTACs remains a considerable challenge. Here, we present an updated online database, PROTAC-DB 3.0. In this third version, we have expanded the database to include 6111 PROTACs (87% increase compared to the 2.0 version). Additionally, the database now contains 569 warheads (small molecules targeting the protein), 2753 linkers, and 107 E3 ligands (small molecules recruiting E3 ligases). The number of target-PROTAC-E3 ternary complex structures has also increased to 959. Recognizing the importance of druggability in PROTAC design, we have incorporated pharmacokinetic data to PROTAC-DB 3.0. To enhance user experience, we have added features for sorting based on molecular similarity and literature publication date. PROTAC-DB 3.0 is accessible at http://cadd.zju.edu.cn/protacdb/.

## Graphical Abstract

**Graphical Abstract:**
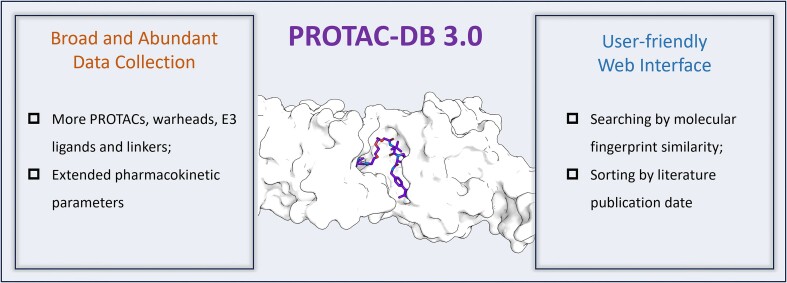

## Introduction

Targeted protein degradation (TPD) has emerged as a significant therapeutic strategy over the past two decades, with proteolysis-targeting chimeras (PROTACs) being a mainstream class of molecules in this field (1–3). PROTACs are heterobifunctional molecules that consist of two ligands connected by a linker. One ligand, known as the warhead, binds to the protein of interest (POI), while the other ligand, called the E3 ligand, recruits an E3 ubiquitin ligase (1). Upon simultaneously binding to both the POI and the E3 ligase, PROTACs induce the ubiquitination of the POI, directing it towards the ubiquitin-proteasome pathway for degradation. The mechanism of action for PROTACs is event-driven rather than occupancy-driven, implying that PROTACs do not require high affinity, large doses to be effective, or targeting the active site of the protein to be effective (4). Consequently, PROTAC technology can be utilized to degrade many targets that are traditionally considered undruggable (5).

Since the first PROTAC molecule was developed in 2001 (1), numerous molecules based on the concept of TPD have been designed. Given that PROTACs were the pioneering idea in this field, many PROTAC molecules have advanced to the clinical development stage (6–8). Examples include ARV-110 (9), which targets androgen receptors (AR), and NX-2127 (10), which targets Bruton's tyrosine kinase (BTK). These molecules in the clinical development stage show potential for treating a variety of hematologic tumors, solid tumors, and other diseases (6,7,9–12).

Although the remarkable efficacy of PROTACs has made them a rising star in the pharmaceutical industry, there are still significant challenges in their rational design. Due to their high molecular weight, PROTACs do not conform to the conventional drug properties of oral drugs, specifically Lipinski's ‘Rule of 5’ (13). Moreover, the advancement and application of deep learning and artificial intelligence technologies in drug discovery have introduced a number of software tools that accelerate the design of PROTACs (14–18). Both the rational design of PROTACs and the development of computational models heavily rely on sufficient data support. Currently, many studies employed data-driven deep learning (DL) technology to assist in the design of PROTAC drugs. For instance, Li *et al.* predicted PROTAC degradation activity based on protein binding pocket information and PROTAC chemical properties (19), Zheng *et al.* designed PROTACs by learning the distribution of linker chemical space and generating models, which were later validated through wet-lab experiments (16). Tang *et al.* utilized molecular dynamics simulations to characterize PROTAC dissociation pathways and binding features (15).

To address this need, we previously released PROTAC-DB and its updated version, PROTAC-DB 2.0 (20,21). These databases provide users detailed information on PROTAC molecules, warheads, linkers and E3 ligands, with associated 2D and 3D structural data and activity data. Since 2020, the database has been operating stably for four years, attracting over 126 000 visits to the database websites. Additionally, the database has been extensively cited by researchers. Similar to PROTAC-DB, several other databases also record PROTAC-related information, such as PROTACpedia (https://protacpedia.weizmann.ac.il/ptcb/main), which currently covers information on more than a thousand PROTACs. In addition to databases specifically related to PROTAC molecules, there are also databases that focus on E3 ligases, such as ELIOT (22), which contains E3 ligase and its corresponding ligands, and UbiHub (23), which is centered around ubiquitination-related information.

Here, we present an updated database, PROTAC-DB 3.0, which offers substantial enhancements in data volume compared to the previous version. For instance, the number of PROTACs has significantly increased from 3270 to 6111. For data-driven methods like DL or machine learning (ML), having assess to more data allows the model to explore a larger chemical space and reduces potential risks, such as overfitting, which can occur due to limited data. Considering that druggability is a crucial factor in the rational design of PROTACs (13), we have also included the pharmacokinetic parameters of these molecules. For the design of PROTACs in animal experiments or clinical stages, pharmacokinetic parameters are crucial. Expanding the collection of such data could assist researchers in designing PROTAC molecules with improved druggability. Additionally, we have introduced functionalities for searching by molecular fingerprint similarity and sorting by literature publication date to facilitate user retrieval.

## Materials and methods

### Data collection and processing

Consistent with our method for collecting data in PROTAC-DB 1.0 and 2.0, we used the keywords ‘degrader* OR protac OR proteolysis targeting chimera’ to search for original papers in the PubMed database (20,21). For each article, we manually collected and recorded the targets, molecular structures and activity information of PROTACs, including the corresponding warheads, linkers, and E3 ligands. Notably, we have added pharmacokinetic parameters in this update. For modeling the ternary complex structures of POI-PROTAC-E3 ligase, several computational tools have been developed for PROTAC modeling, such as ProsettaC, PROTAC-Model, and others (14,17,18,24). These tools rely on protein-protein interactions (PPIs) conformations to sample the conformations of PROTAC molecules. Traditionally, modeling basic PPI conformations involves using PPI docking software. However, with the advent of AlphaFold-Multimer (25), it is now feasible to predict PPI conformations directly from sequences. In this work, we chose to use PROTAC-Model to construct the ternary complex, as it provides superior prediction results based on unbound structures. PROTAC-Model integrates FRODOCK (26) for local docking, RosettaDock (27) for structural optimization, and RDKit (an open-source cheminformatics, https://www.rdkit.org) for PROTAC molecule sampling.

### Pharmacokinetic parameters collection

Pharmacokinetic parameters describe the processes of absorption, distribution, metabolism, and excretion of a drug in living organisms, and are a crucial component of both preclinical and clinical drug research. In PROTAC-DB 3.0, we have collected and presented the pharmacokinetic parameter entries in Table 1.

**Table 1.** The abbreviations and full forms of pharmacokinetic parameters in PROTAC-DB 3.0

Abbreviations	Full forms
Tmax	Time to reach maximum concentration
T1/2	The half-life
Cmax	Maximum concentration
AUC (0-t)	Area under the plasma concentration-time curve from time 0 extrapolated to quantifiable time
AUC (0-infinity)	Area under the plasma concentration-time curve from time 0 extrapolated to infinite time
Vz	The volume of distribution
Vz/F	Apparent volume of distribution
Vss	The volume in steady state
CL	Clearance
CL/F	Apparent clearance
MRT (0-t)	Mean residence time from time 0 extrapolated to quantifiable time
MRT (0-infinity)	Mean residence time from time 0 extrapolated to infinite time
F	Bioavailability

### Molecular fingerprint similarity calculation

To calculate molecular similarity based on SMILES for retrieval, we first converted all molecules, including PROTACs, warheads, and E3 ligands, into Morgan fingerprint representations. This was done using the RDKit function ‘GetMorganFingerprint’ with a radius parameter set to 2, creating a molecular fingerprint library for search. For each query molecule, we generated a molecular fingerprint in the same process and calculated the Tanimoto similarity using the RDKit function ‘TanimotoSimilarity’. The resulting similarity values were then used to rank the search results.

## Results

### Data overview

**Figure 1.**
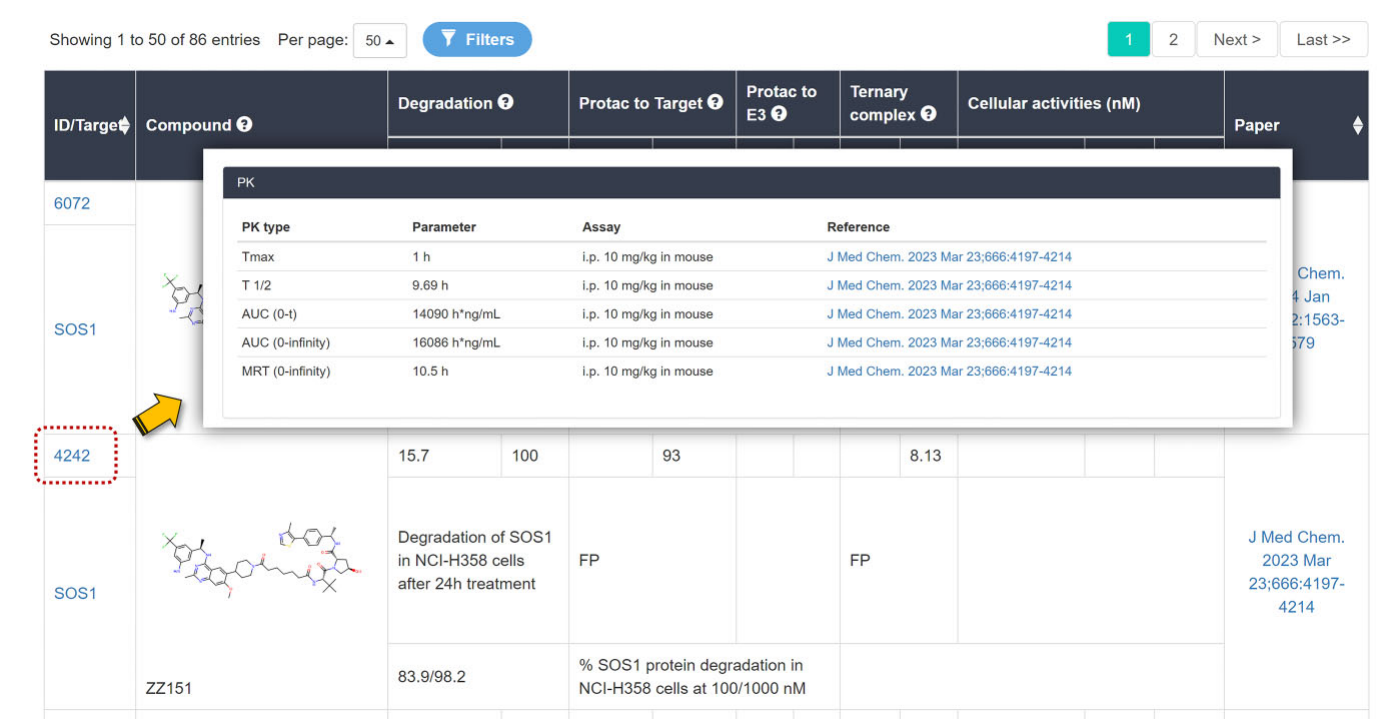
Pharmacokinetic parameter tabs in the detailed information pages of PROTACs.

Over recent years, the number of PROTAC molecules has increased significantly. Here we have updated the PROTAC-DB database, and the detail information of the update are summarized in Table 2. In PROTAC-DB 3.0, the number of PROTAC molecules was updated from 3270 to 6111 (an increase of about 87%). Meanwhile, the number of warheads increased from 365 to 569, E3 ligands from 82 to 107, and linkers from 1501 to 2753. PROTAC-DB 3.0 also updates a variety of biological activity data, including DC_50_ data (from 705 to 1308), cellular activity data (from 1095 to 1871), Western blotting data (from 2073 to 2988). In addition, the binding affinity data between PROTACs and target proteins increased from 818 to 1251, between PROTACs and E3 ligases increased from 198 to 229, and for the formation of ternary complexes increased from 54 to 73. As for the statistics of POI and E3 ligases, PROTAC-DB 3.0 recorded 442 and 20 classes of proteins (280 and 13 in PROTAC-DB 2.0), respectively. Moreover, the number of the 3D structures of ternary complexes has also increased significantly, with the crystal structures increasing from 18 to 23 and the predicted structures from 664 to 959. Regarding druggability parameters, PROTAC’s cell permeability data increased from 41 to 64 entries. Additionally, in this update, we have added pharmacokinetic parameter information, totaling 145 items. The pharmacokinetic parameter information is displayed in a separate tab on the detailed information pages of PROTACs, as shown as Figure 1.

**Table 2.** Data statistics of PROTAC-DB 1.0, 2.0 and 3.0

Data category	Version 1.0	Version 2.0	Version 3.0
Number of PROTACs	1662	3270	6111
Number of warheads	202	365	569
Number of E3 ligands	65	82	107
Number of linkers	806	1501	2753
Number of PROTACs with DC50 data	379	705	1308
Number of PROTACs with cellular activity data	437	1095	1871
Number of PROTACs with Western blotting data	1144	2073	2988
Number of PROTACs with binding affinity data between PROTACs and target proteins	411	818	1251
Number of PROTACs with binding affinity data between PROTACs and E3 ligases	130	198	229
Number of PROTACs with binding affinity data for the formation of ternary complexes	26	54	73
Number of target proteins	147	280	442
Number of E3 ligases	11	13	20
Number of crystal structures	11	18	23
Number of predicted structures	/	664	959
Number of PROTACs with cell permeability data	/	41	64
Number of PROTACs with pharmacokinetics data	/	/	145

### Searching and sorting based on molecular similarity

**Figures 2.**
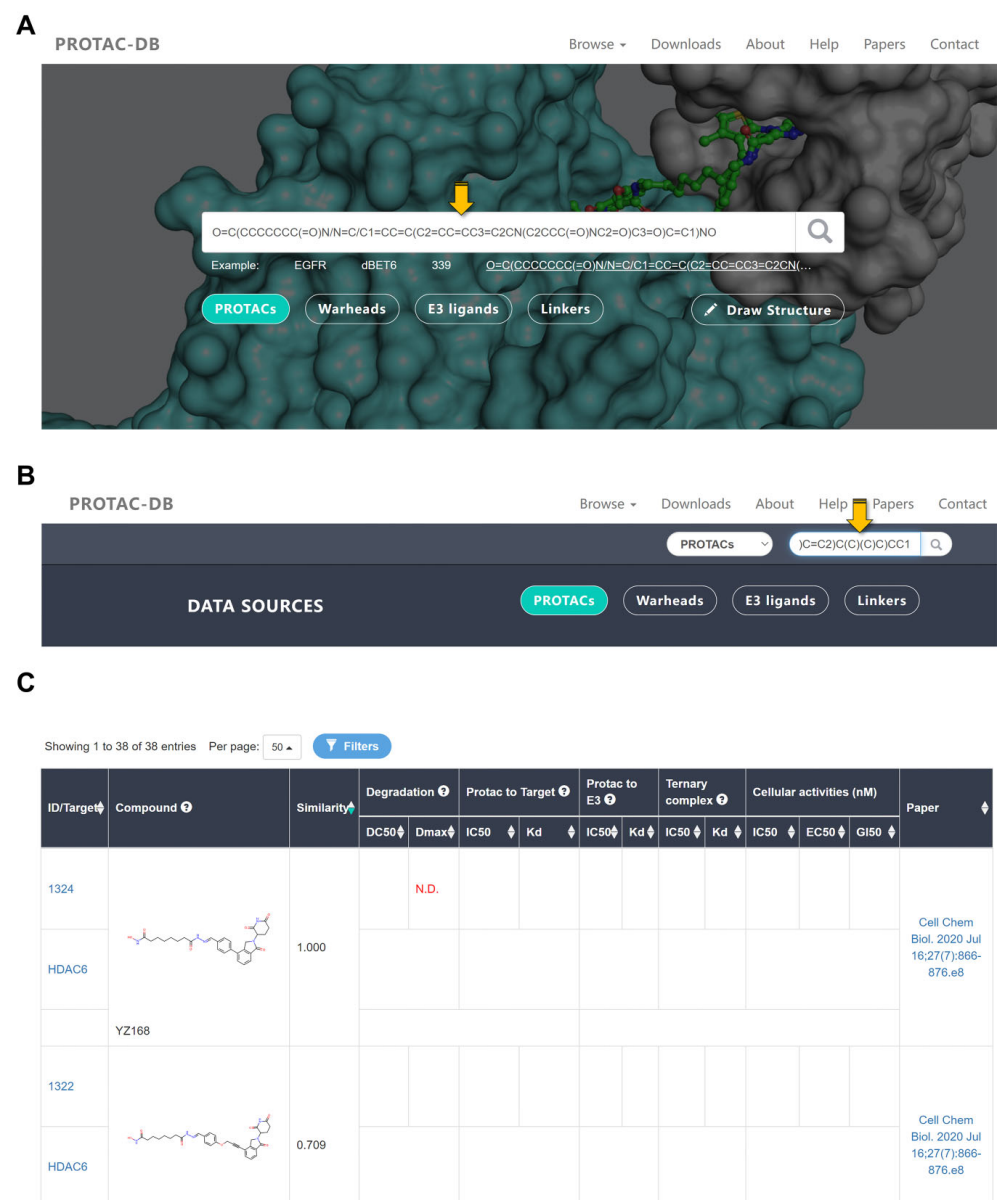
(**A**) and (**B**) demonstrate the use of the search bar on both the main page and the browse page. (**C**) shows the results of the search sorted by molecular similarity.

In the server of PROTAC-DB 3.0, we have incorporated a feature that allows users to directly use SMILES strings for searching, thereby enhancing user accessibility. As illustrated in Figure 2A and B, users can use the search bar on the Main Page or the browse page to search for ‘PROTACs’, ‘Warheads’, ‘E3 ligands’, or ‘Linkers’. This feature transforms the SMILES string into a Morgan fingerprint, a representation similar to Functional-Class Fingerprint (FCFP). It iteratively compiles the atomic environment into a molecular fingerprint and then organizes the results based on Tanimoto similarity. The detailed information on the conversion process and result calculation can be found in the Materials and methods section. An illustrative search result is depicted in Figure 2C. Similar to biological activity data, users have the option to sort the results from low to high or vice versa based on Tanimoto similarity. We have established a minimum threshold of 0.5, so the search page will only display results with molecular similarity exceeding this value. Similarly, searches can also be conducted for warheads and E3 ligands.

### Sorting based on literature publication date

**Figures 3.**
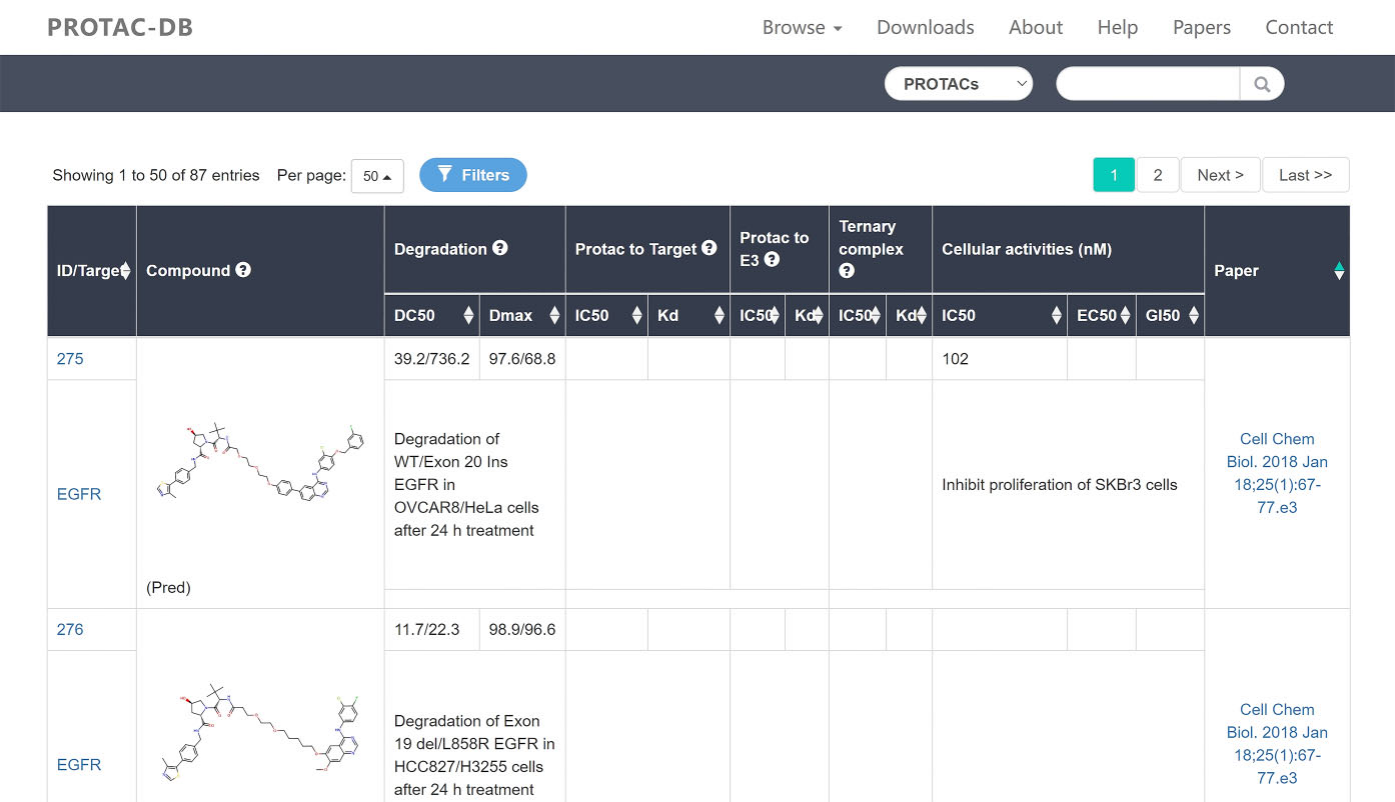
Search page results are sorted on literature publication date.

In PROTAC-DB 2.0, our page only displays references for PROTAC molecules. However, in the PROTAC-DB 3.0 update, we have introduced the release date of references as a sorting option. Users can now sort the molecules by their release date after searching (as shown in Figure 3). This enhancement is designed to facilitate the search and browsing processes for drug designers.

## Conclusion

PROTAC-guided targeted protein degradation technology has revolutionized drug design, offering new solutions for previously difficult-to-target molecules. The integration of artificial intelligence in drug design has further accelerated the development of PROTACs. However, the technology remains in its developmental stages and requires substantial reliable data to advance. PROTAC-DB continues to receive updates and support, now including 6111 PROTAC molecules in version 3.0 (up from 3270 in version 2.0). This significant increase in data volume enhances the database's utility. Given the challenges associated with the druggability of PROTACs, we have also increased the inclusion of pharmacokinetic data, currently encompassing 145 relevant entries. To improve user experience, we have added features for sorting by molecular similarity and literature publication date. These enhancements are expected to make PROTAC-DB 3.0 a more valuable resource for the rational design of PROTACs.

## Data Availability

PROTAC-DB 3.0 is accessible at http://cadd.zju.edu.cn/protacdb/.
